# Nore1 inhibits age-associated myeloid lineage skewing and clonal hematopoiesis but facilitates termination of emergency (stress) granulopoiesis

**DOI:** 10.1016/j.jbc.2023.104867

**Published:** 2023-05-27

**Authors:** Olatundun Williams, Liping Hu, Weiqi Huang, Priyam Patel, Elizabeth T. Bartom, Ling Bei, Elizabeth Hjort, Christina Hijiya, Elizabeth A. Eklund

**Affiliations:** 1 Vagelos College of Physicians and Surgeons, Columbia University Irving Medical Center, New York, New York, USA; 2 The Feinberg School of Medicine, Northwestern University, Chicago, Illinois, USA; 3 Robert H. Lurie Comprehensive Cancer Center, Northwestern University, Chicago, Illinois, USA; 4 Medicine Service, Jesse Brown VA Medical Center, Chicago, Illinois, USA; 5 RxD Nova Pharmaceuticals, Inc, Vacaville, California, USA; 6 Cognition Studios, Inc, Seattle, Washington, USA; 7 Yale School of Public Health, Yale University, New Haven, Connecticut, USA

**Keywords:** myeloid leukemia, innate immune response, apoptosis, gene regulation, hematopoiesis

## Abstract

Age-associated bone marrow changes include myeloid skewing and mutations that lead to clonal hematopoiesis. Molecular mechanisms for these events are ill defined, but decreased expression of Irf8/Icsbp (interferon regulatory factor 8/interferon consensus sequence binding protein) in aging hematopoietic stem cells may contribute. Irf8 functions as a leukemia suppressor for chronic myeloid leukemia, and young *Irf8*^−/−^ mice have neutrophilia with progression to acute myeloid leukemia (AML) with aging. Irf8 is also required to terminate emergency granulopoiesis during the innate immune response, suggesting this may be the physiologic counterpart to leukemia suppression by this transcription factor. Identifying Irf8 effectors may define mediators of both events and thus contributors to age-related bone marrow disorders. In this study, we identified *RASSF5* (encoding Nore1) as an Irf8 target gene and investigated the role of Nore1 in hematopoiesis. We found Irf8 activates *RASSF5* transcription and increases Nore1a expression during emergency granulopoiesis. Similar to *Irf8*^−/−^ mice, we found that young *Rassf5*^−/−^ mice had increased neutrophils and progressed to AML with aging. We identified enhanced DNA damage, excess clonal hematopoiesis, and a distinct mutation profile in hematopoietic stem cells from aging *Rassf5*^−/−^ mice compared with wildtype. We found sustained emergency granulopoiesis in *Rassf5*^−/−^ mice, with repeated episodes accelerating AML, also similar to *Irf8*^−/−^ mice. Identifying Nore1a downstream from Irf8 defines a pathway involved in leukemia suppression and the innate immune response and suggests a novel molecular mechanism contributing to age-related clonal myeloid disorders.

Myeloid skewing and hematopoietic stem cell (HSC) expansion are found in the bone marrow of some aging humans and in mice (1). In human subjects, this may be associated with mutations in various leukemia-associated genes, defining “clonal hematopoiesis of indeterminate potential” (CHIP) if mutations are present with a variant allelic frequency (VAF) of greater than 2% (1). Molecular mechanisms for such bone marrow changes are undefined, but recent transcriptome profiling of HSCs from aging humans or mice suggests possibilities (2, 3, 4, 5). For example, these studies defined an age-related decrease in expression of interferon regulatory factor 8 (Irf8) (also referred to as interferon consensus sequence binding protein [Icsbp]). This is of interest since the net effect of this transcription factor is to inhibit proliferation, enhance apoptosis, facilitate DNA repair, and modulate phagocyte effector functions during myeloid differentiation (6, 7, 8, 9, 10, 11, 12).

Relatively decreased expression in the bone marrow of subjects with chronic myeloid leukemia (CML) compared with nonleukemic subjects suggests that Irf8 functions as a leukemia suppressor (13). In CML, expression of Irf8 increases with remission, decreases with relapse, and is lowest during progression to myeloid blast crisis (BC or acute myeloid leukemia [AML]) (13). Consistent with this, leukemogenesis is delayed in mice transplanted with bone marrow cotransduced with vectors to express both Bcr-abl and Irf8 compared with recipients of bone marrow–expressing Bcr-abl alone (14, 15). *Irf8*^−/−^ mice phenocopy CML with neutrophilia at a young age and development of AML over time (16, 17).

We found Irf8 is also required to terminate emergency (stress) granulopoiesis; the episodic process for neutrophil production during the innate immune response (18, 19). Emergency granulopoiesis is mechanistically distinct from steady-state granulopoiesis, involving different cytokines and regulatory pathways (20, 21). During episodes of emergency granulopoiesis, we found exaggerated and sustained neutrophilia in *Irf8*^−/−^ mice compared with wildtype (18, 22). Repeated challenges accelerated progression to AML in *Irf8*^−/−^ mice but had no adverse effects on wildtype mice (18). Emergency granulopoiesis was stimulated by activation of the Nlrp3 inflammasome in these studies. In mice with Bcr-abl-induced CML, we found similar dysregulation of neutrophil production during repeated episodes of emergency granulopoiesis with acceleration of chronic phase relapse in mice in tyrosine kinase inhibitor–induced remission and enhanced progression to BC (19).

We previously described a set of Irf8 target genes that contribute to leukemia suppression and termination of emergency granulopoiesis (6, 7, 8, 9, 10, 11, 12). This includes *PTPN13* (encoding Fas-associated phosphatase 1, a Fas inhibitor), *GAS2* (encoding growth arrest specific 2, a calpain inhibitor), *CAST* (encoding calpastatin, a calpain inhibitor), *NF1* (encoding neurofibromin 1, a Ras antagonist), *FANCC* and *FANCF* (encoding Fanconi DNA-repair proteins), and *CYBB* and *NCF2* (encoding gp91^phox^ and p67^phox^, phagocyte NADPH-oxidase proteins). Tyrosine phosphorylation and interaction of Irf8 with various partner proteins regulates repression or activation of target genes at various stages of myelopoiesis (11, 13, 23). Using a chromatin immunoprecipitation–based screen, we also identified *RASSF5*, encoding Nore1, as a putative Irf8 target gene (6). Consistent with this, publicly available databases defined a decrease in Nore1 mRNA in CML bone marrow compared with control subjects (https://www.oncomine.org). In the current study, we investigate the relationship between Irf8 and Nore1 and the contribution of Nore1 to regulation of hematopoiesis during aging and emergency granulopoiesis.

The *RASSF5* gene produces Nore1a and b isoforms through use of different promoters, and we identified Irf8 binding to the A promoter (24, 25, 26). Nore1a is a ubiquitously expressed protein with an N-terminal protein kinase C–conserved region (C1), a Ras association (RA) domain, and a C-terminal SARAH domain (25, 26, 27, 28). Nore1b expression is restricted to activated T cells and contains RA and SARAH domains but lacks the N-terminal C1 domain (25, 26, 27, 28). Like other RA domain family (Rassf) proteins, Nore1 is a scaffold for multiprotein complexes, influencing cell function by facilitating protein–protein interactions (29, 30, 31, 32). For example, Nore1 directs the Mst1, a serine/threonine kinase, to the cell membrane where it autophosphorylates and enhances activation of caspases and/or stress-activated protein kinases during Fas or tumor necrosis factor alpha (TNFα)–induced apoptosis (29, 30). This suggests that *RASSF5* transcription may mediate some effects of Irf8 on apoptosis.

*Rassf5*^*−/−*^ mice lack expression of both Nore1a and b isoforms but have no obvious phenotype. Hepatocytes from these mice are resistant to TNFα or TRAIL-induced apoptosis and fail to activate Mst1 *in vivo* (25). In some human solid tumors, *RASSF5* deletion or silencing correlates with aggressive disease (33, 34, 35, 36, 37, 38, 39, 40, 41, 42, 43, 44, 45). However, hematopoiesis in *Rassf5*^*−/−*^ mice was not previously studied, and implications for leukemogenesis are unknown. In the current work, we investigate Nore1 as a mediator of leukemia suppression, emergency granulopoiesis termination, and age-associated clonal hematopoiesis. Events downstream from Irf8 and Nore1a may clarify the contribution of infectious episodes to constitutive activation of inflammatory pathways and clonal hematopoiesis during aging and suggest therapeutic targets to mitigate such effects.

## Results

### Irf8 activates *RASSF5* promoter A and enhances Nore1a expression

**Figure 1:**
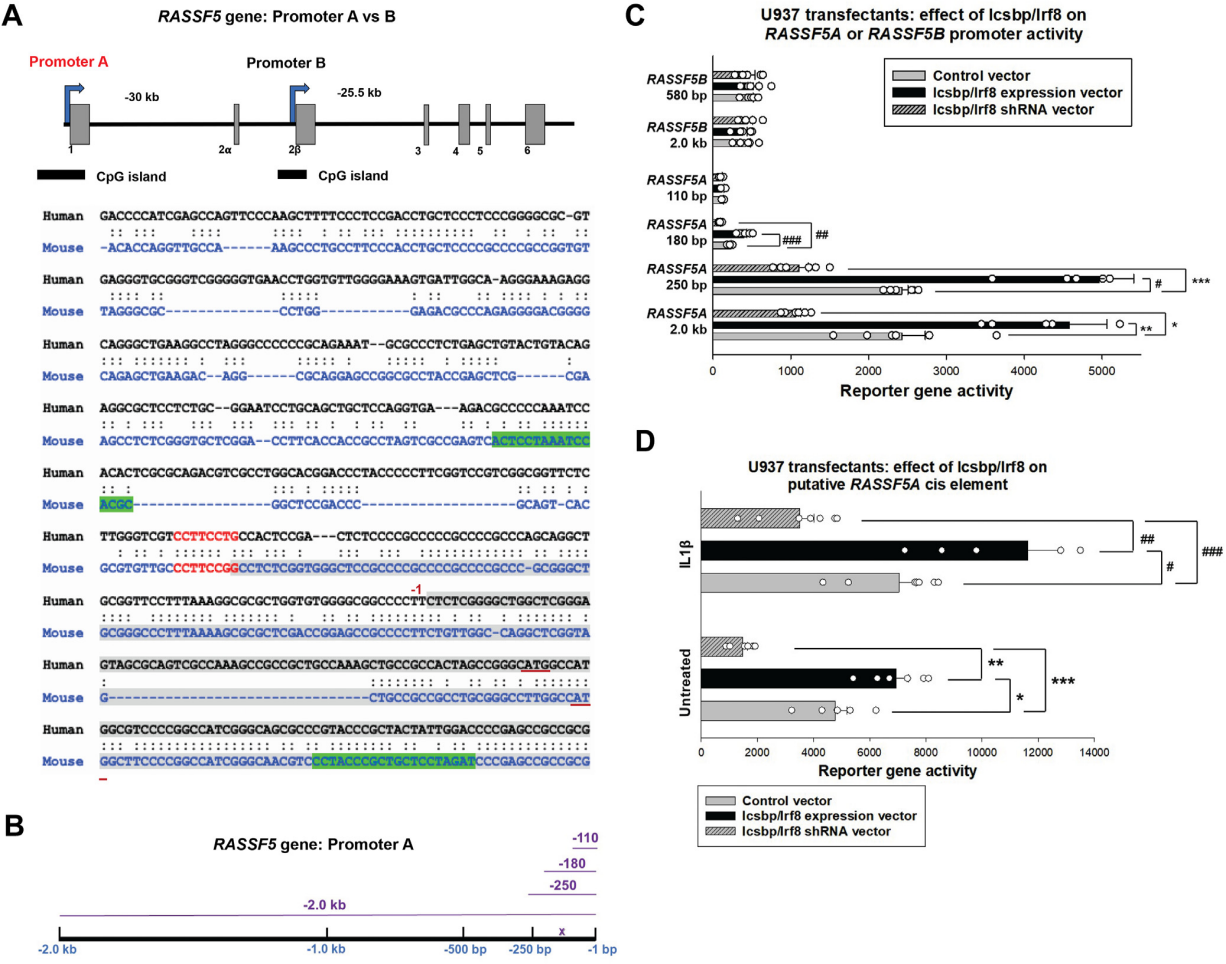
**Irf8 activates *RASSF5* promoter A.***A*, a CpG island in *RASSF5* promoter A was identified by a chromatin immunoprecipitation–based screen with an antibody Irf8 (also referred to as Icsbp) (6). The *RASSF5A* promoter was analyzed for potential Irf8-binding sequences, and a conserved Ets/Irf consensus is indicated in *red*. Primers for chromatin immunoprecipitation studies are in *green*. Exon 1 is indicated in *gray*, and the ATG codon for translation start is underlined in *red*. *B*, *cartoon* depiction of *RASSF5* promoter A sequences used analyzed in reporter gene assays. Constructs are indicated by *purple lines* and the conserved Ets/Irf consensus by a *purple* X. *C*, Irf8 activates *RASSF5* promoter A constructs with at least 180 bp of 5′ flank. U937 cells were transfected with *RASSF5* promoter A/luciferase reporter constructs or *RASSF5* promoter B/reporter constructs. Cells were cotransduced with vectors to overexpress or knockdown Irf8 (or with relevant control vectors). Each construct was analyzed in at least five independent transfection experiments. Statistical significance is indicated by ∗ (*p* = 0.002), ∗∗ (*p* = 0.003), ∗∗∗ (*p* = 0.0004), # (*p* = 0.0002), ## (*p* = 0.0005), or ### (*p* = 0.0003). Error bars represent SD, and *open circles* represent individual data points. The empty reporter vector was not influenced by overexpression or knockdown of Irf8, and this minimal activity was subtracted as background. *D*, Irf8 activates a conserved Ets/Irf consensus sequence between 180 bp and 110 bp in *RASSF5* promoter A. U937 cells were transfected with a reporter construct with a minimal promoter and three copies of the Ets/Irf consensus sequence from the A promoter and vectors to overexpress or knockdown Irf8. Some cells were stimulated with interleukin 1β (IL1β) for 24 h prior to analysis. Each construct was analyzed in at least five independent experiments. Statistical significance for panels in this figure is indicated by ∗ (*p* = 0.004), ∗∗ (*p* = 0.005), ∗∗∗, # (*p* = 0.004), ## (*p* = 0.0004), or ### (*p* = 0.01). Error bars represent SD, and *open circles* represent individual data points. The empty reporter vector was not influenced by overexpression or knockdown of Irf8, or treatment with IL1β and this minimal activity was subtracted as background. Icsbp, interferon consensus sequence binding protein; Irf8, interferon regulatory factor 8.

To identify target genes that mediate effects of Irf8 (referred to as Icsbp in prior studies), we hybridized chromatin that coimmunoprecipitated from U937 myeloid leukemia cells with Irf8 to a CpG island microarray (6). U937 cells undergoing cytokine-induced differentiation were studied to identify Irf8 target genes associated with the innate immune response. We previously reported functional characterization of several genes that were identified in these studies (6, 7, 8, 9, 10). Further examination of these data identified interaction between Irf8 and a CpG island in the *RASSF5A* promoter (Fig. 1*A*).

To determine if Irf8 functionally impacted the *RASSF5A* promoter, U937 myeloid cells were transfected with series of reporter constructs with promoter A truncations (Fig. 1*B*) and vectors to overexpress Irf8 or express Irf8-specific shRNAs *versus* relevant control vectors. We found that activity of reporter constructs with 2 kb, 250 bp, or 180 bp of promoter A was increased by Irf8 overexpression (∼70% increase by Irf8 with each of these constructs, *p* < 0.01, n = 10), but a 110 bp construct was not (*p* = 0.6, n = 10) (Fig. 1*C*). In contrast, Irf8 knockdown decreased reporter activity of the constructs to below the activity seen without knockdown (∼65% decrease, *p* < 0.01, n = 5 for Irf8-specific shRNAs *versus* control). This is consistent with the location of a conserved Ets/Irf consensus sequence identified between −180 and −110 bp in the human promoter. In these experiments, neither overexpression nor knockdown of Irf8 influenced activity of the control reporter vector (without *RASSF5A* promoter sequences), and this was subtracted as background.

We generated another reporter construct with three copies of this promoter A Ets/Irf-consensus sequence linked to a minimal promoter. We found Irf8 overexpression increased, but Irf8-specific shRNAs decreased, activity of this construct relative to control vectors that did not either overexpress or knockdown Irf8 (*p* ≤ 0.004, n = at least 5) (Fig. 1*D*). Since binding of Irf8 to the *RASSF5A* promoter was identified in studies with differentiating U937 cells, we investigated the impact of interleukin 1β (IL1β) on this *cis* element. IL1β is the major mediator of Nlrp3 inflammasome–induced emergency granulopoiesis (22, 46). We found that *cis* element activity increased significantly with IL1β, with or without Irf8 overexpression or knockdown (∼60% increase under all conditions, *p* ≤ 0.01, n = at least 5 for comparison to control vectors without Irf8 overexpression or knockdown). Control experiments were performed with the minimal promoter/reporter vector (without the *RASSF5A cis* element). Inclusion of the putative *cis* element increased reporter expression above the control vector alone. Neither Irf8 overexpression nor Irf8 knockdown altered expression of the control minimal promoter/reporter construct, and this was subtracted as background.

As a control, we also investigated the effect of Irf8 on reporter constructs with the proximal 2.0 kb or 580 bp of *RASSF5* promoter B. Activity of promoter B constructs was significantly less than A, consistent with restriction of Nore1b expression to T lymphocytes (Fig. 1*C*). Compared with control expression vector, neither Irf8 overexpression (*p* = 0.3 and *p* = 0.5, n = 4 for the 2.0 kb and 580 bp constructs, respectively) nor knockdown of Irf8 (*p* = 0.6 and *p* = 0.4, n = 4 for the 2.0 kb and 580 bp construct) influenced activity of *RASSF5B* constructs.

We next investigated the impact of Irf8 on Nore1 expression *in vivo*. Since Irf8 is required to terminate emergency granulopoiesis, we determined if it influenced expression of Nore1a during this process. For these studies, we injected *Irf8*^−/−^ or wildtype mice with alum to induce emergency granulopoiesis (*via* Nlrp3 inflammasome activation) or saline as a steady-state control (18, 19, 22). Bone marrow was harvested 2 weeks later, representing the peak abundance of neutrophils in the circulation and bone marrow in alum-injected wildtype mice. We quantified Nore1 mRNA in Lin^−^ or Lin^+^ bone marrow cells with primers specific to Nore1a or Nore1b.

**Figure 2:**
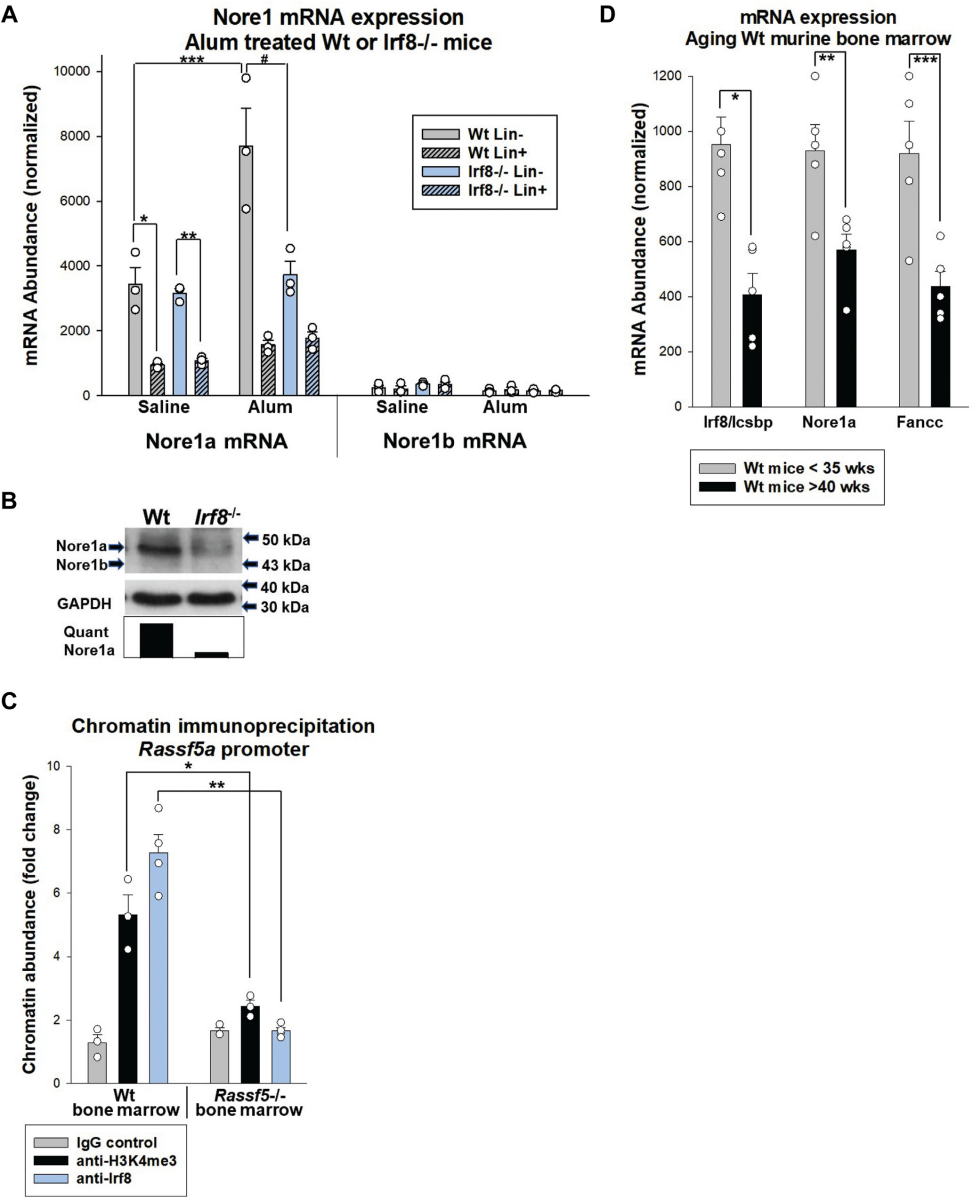
**Irf8 enhances Nore1a expression during emergency granulopoiesis and aging.** Bone marrow cells were isolated from *Irf8*^−/−^ or wildtype mice under various conditions. *A*, Nore1a mRNA increases in Lin^−^ cells from wildtype mice during emergency granulopoiesis but not Lin^−^ cells from *Irf8*^−/−^ mice. Mice were injected with alum to induce emergency granulopoiesis or saline as steady-state control and sacrificed 2 weeks later. Lin^−^ and Lin^+^ cells were isolated, and expression of Nore1a and Nore1b mRNA was quantified by real-time PCR. Statistical significance is indicated by ∗ (*p* = 0.003), ∗∗ (*p* = 0.006), ∗∗∗ (*p* = 0.04), or # (*p* = 0.005), and each sample was analyzed in at least three independent experiments. Error bars represent SD, and *open circles* represent individual data points. *B*, *Irf8*^−/−^ bone marrow progenitor cells express less Nore1a compared with wildtype cells. Murine bone marrow Lin^−^ckit^+^ cells were stimulated with interleukin 1β (IL1β) *ex vivo* and analyzed by Western blots serially probed with antibodies to Nore1 and GAPDH (as a loading control). *C*, Irf8 and Histone 3 (K4me3) immunoprecipitate *Rassf5* promoter A from wildtype, but not *Irf8*^−/−^, murine bone marrow cells. Lin^−^ cells were isolated from alum-injected mice, and immunoprecipitating chromatin was quantified by real-time PCR with primers flanking the Ets/Irf consensus. Data represent fold increase relative to isotype control antibody, and statistical significance is indicated by ∗ (*p* = 0.002, n = 3) or ∗∗ (*p* = 0.001, n = 3). Error bars represent SD, and *open circles* represent individual data points. *D*, expression of Irf8, Nore1a, and Fancc genes decreases with aging. Lin^−^ckit^+^ bone marrow cells from wildtype mice <35 weeks or >40 weeks old were compared, and mRNA expression was quantified by real-time PCR. Statistically significant differences are indicated by ∗ (*p* = 0.02), ∗∗ (*p* = 0.02), or ∗∗ (*p* = 0.01). Each experiment was repeated at least three times. Error bars represent SD, and *open circles* represent individual data points. Irf8, interferon regulatory factor 8.

In both *Irf8*^−/−^ and wildtype bone marrow, we found Nore1a mRNA was more abundant in Lin^−^ *versus* Lin^+^ cells, with or without alum injection (Fig. 2*A*). At steady state, expression of Nore1a mRNA was equivalent in the two genotypes in Lin^−^ or Lin^+^ cells (*p* ≥ 0.1, n = 3 for all comparisons). In wildtype Lin^−^ cells, Nore1a mRNA increased significantly 2 weeks after alum injection (∼2-fold, *p* = 0.04, n = 3), but this increase was not observed in *Irf8*^−/−^Lin^−^ cells (*p* = 0.2, n = 3) (Fig. 2*A*). Therefore, Nore1a expression was significantly impaired in *Irf8*^−/−^Lin^−^ cells relative to wildtype during emergency granulopoiesis (*p* = 0.005, n = 3).

In control experiments, we determined that Nore1b was expressed at lower levels compared with Nore1a in Lin^−^ and Lin^+^ bone marrow cells. Nore1b was not altered by Irf8 knockout or alum injection, consistent with known T cell-restricted expression (Fig. 2*A*). For these studies, relative expression of Nore1a *versus* Nore1b was determined by normalization to a known quantity of plasmid containing the Nore1a complementary DNA (cDNA).

We also investigated Nore1 expression in murine bone marrow myeloid progenitor cells (Lin^−^ckit^+^) after *in vivo* stimulation with IL1β. We found less Nore1a protein in *Irf8*^−/−^ cells compared with wildtype cells (by Western blot), consistent with mRNA expression. Isoforms were identified by size, and these cells did not express Nore1b (Fig. 2*B*).

To verify interaction of Irf8 with *RASSF5* promoter A in murine bone marrow, we performed chromatin immunoprecipitation with Lin^−^ bone marrow cells from wildtype or *Irf8*^−/−^ mice. Since Irf8 binds to a composite Ets/Irf consensus sequences in many promoters, we quantified coprecipitating chromatin with primers flanking the conserved Ets/Irf composite in promoter A (consensus indicated in *red* and primers highlighted in *green*) (Fig. 1*A*). We found enrichment of this region in chromatin that immunoprecipitated with antibody to Irf8 (*p* = 0.001, n = 3 *versus* control antibody) or trimethyl K4-histone 3 (*p* = 0.002, n = 3 *versus* control antibody), consistent with activation by Irf8 (Fig. 2*C*) (47). *Irf8*^−/−^ murine bone marrow cells were a negative control for this experiment.

Decreased Irf8 expression in aging human and murine bone marrow HSCs was previously documented (2, 3). Therefore, we determined the impact of aging on expression of Irf8 and select target genes in Lin^−^ bone marrow cells from young *versus* aging wildtype mice (<30 weeks *versus* >40 weeks) (Fig. 2*D*). We found decreased Irf8 in aging mice, associated with decreased expression of Irf8 activation target genes, Nore1a and Fancc (*p* < 0.02, n = 3 for all three genes) (46). We previously determined that Irf8-enhanced expression of Fanconi C during emergency granulopoiesis is essential to handle genotoxic stress of this process (46).

### Apoptosis-resistant HSCs expand in aging *Rassf5*^−/−^ mice

**Figure 3:**
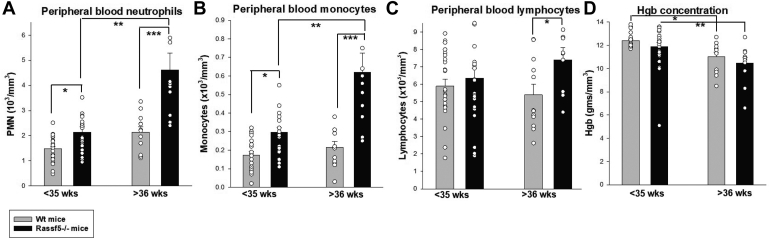
**Aging *Rassf5***^**−/−**^**mice exhibit neutrophilia and monocytosis.** Peripheral blood counts were determined in young (<35 weeks of age) or aging (>36 weeks of age) *Rassf5*^−/−^ and wildtype mice. *A*, *Rassf5*^−/−^ mice have relative peripheral blood neutrophilia compared with wildtype mice. Statistically significant difference is indicated by ∗ (*p* = 0.003), ∗∗ (*p* = 0.0006), or ∗∗∗ (*p* = 0.004). *B*, *Rassf5*^−/−^ mice exhibit significant monocytosis compared with wildtype mice. Statistically significant difference is indicated by ∗ (*p* = 0.01), ∗∗ (*p* = 0.003), or ∗∗∗ (*p* = 0.01). *C*, total circulating lymphocytes decrease in aging wildtype mice but not *Rassf5*^−/−^ mice. Statistical significance is indicated by ∗ (*p* = 0.05) or ∗∗ (*p* = 0.04). *D*, progressive anemia occurs with aging in *Rassf5*^−/−^ and wildtype mice. Statistically significant is indicated by ∗ (*p* = 0.0008) or ∗∗ (*p* = 0.03). For each experiment above, n = 9 at least, error bars represent SD, and *open circles* represent individual data points.

To determine the impact of Nore1 on hematopoiesis, we compared peripheral blood counts in *Rassf5*^−/−^ and wildtype mice over time. We found relative neutrophilia and monocytosis in young *Rassf5*^−/−^ mice *versus* wildtype, which further increased during aging (comparison of mice <35 *versus* >36 weeks). Analysis of variance between groups demonstrated a significantly greater age-related increase in circulating neutrophils (Fig. 3*A*) and monocytes (Fig. 3*B*) in *Rassf5*^−/−^ mice compared with wildtype. Aging *Rassf5*^−/−^ mice in these studies had <20% circulating myeloid blasts (discussed further later). Circulating lymphocytes decreased slightly with age in wildtype mice but increased slightly in *Rassf5*^−/−^ mice, resulting in relative lymphocytosis in aging *Rassf5*^−/−^ mice (*p* = 0.03, n = 9 for comparison between genotypes) (Fig. 3*C*). Peripheral blood hemoglobin concentration decreased significantly with age in both *Rassf5*^−/−^ and wildtype mice (*p* = 0.0008, n = 9 and *p* = 0.03, n = 12, respectively) (Fig. 3*D*). Platelet counts were stable over time without differences between genotypes (not shown).

**Figure 4:**
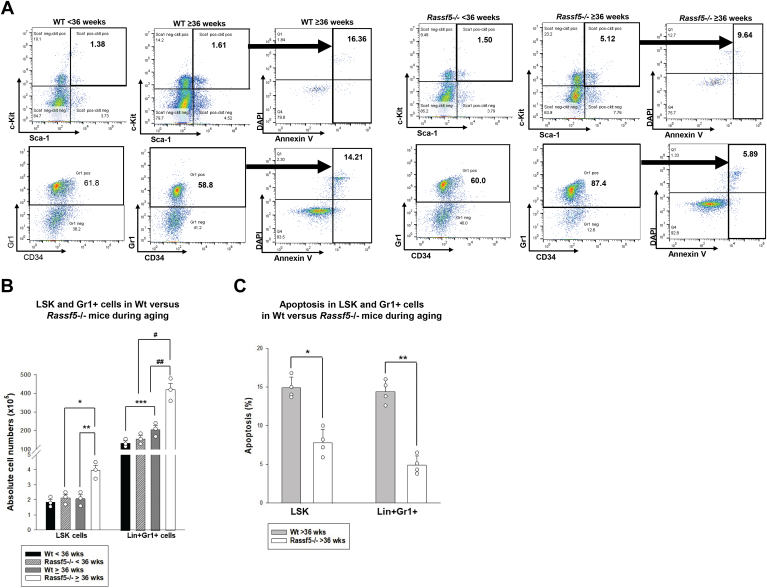
**Myeloid expansion in aging *Rassf5***^**−/−**^**murine bone marrow is associated with apoptosis resistance of hematopoietic stem cells and differentiating granulocyte progenitors.** Bone marrow analysis was performed in young (<36 weeks) or aging (≥36 weeks) *Rassf5*^−/−^ and wildtype mice. *A*, during aging, hematopoietic stem cells and differentiating granulocyte progenitors expand and are relatively apoptosis resistant in the bone marrow of *Rassf5*^−/−^ mice compared with wildtype mice. Population distribution was determined by flow cytometry for Lin^−^Sca1^+^ckit^+^ (LSK) and Lin^+^Gr1^+^ cells (differentiating granulocytes) and apoptosis by Annexin V staining. *B*, quantification of expanded LSK and differentiating granulocytes in *Rassf5*^−/−^*versus* wildtype mice with aging. Statistically significant difference is indicated by ∗ (*p* = 0.01), ∗∗ (*p* = 0.01), ∗∗∗ (*p* = 0.05), # (*p* = 0.002), and ## (*p* = 0.006). *C*, quantification of apoptosis in LSK and Lin^+^Gr1^+^ cells from *Rassf5*^−/−^*versus* wildtype mice during aging. Statistical significance is indicated by ∗ (*p* = 0.04) or ∗∗ (*p* = 0.007). Each experiment was repeated at least three times, error bars in this figure represent SD, and *open circles* represent individual data points.

These results suggest that Nore1 impacts development of phagocytic cells in an age-dependent manner. To investigate further, we analyzed the population distribution of bone marrow cells from these aging *Rassf5*^−/−^ and wildtype mice. In young mice, we found no significant difference in abundance of Lin^−^Sca1^+^ckit^+^ cells (LSK, *p* = 0.2, n = 5), Lin^−^Sca1^−^ckit^+^ cells (LK, granulocyte–monocyte progenitors, *p* = 0.4, n = 5), or Lin^+^Gr1^+^ cells (differentiating and mature granulocytes, *p* = 0.5, n = 5) between *Rassf5*^−/−^ and wildtype mice (Fig. 4*A*). However, LSK and Lin^+^Gr1^+^ cells increased during aging in *Rassf5*^−/−^ mice (*p* < 0.01, n = 5 for both comparisons) (Fig. 4*A*) but not in wildtype mice. Therefore, HSC expansion and age-associated myeloid skewing were relatively greater in *Rassf5*^−/−^ mice.

To define a mechanism for these differences, we studied apoptosis in bone marrow populations from young *versus* aged *Rassf5*^−/−^ and wildtype mice, as defined previously. We found that LSK and Lin^+^Gr1^+^ cells from aged *Rassf5*^−/−^ mice were apoptosis resistant compared with cells from similarly aged wildtype mice (*p* = 0.015, n = 6 for LSK cells, *p* < 0.001, n = 6 for Lin^+^Gr1^+^ cells) (Fig. 4*B*). In young mice, differential apoptosis sensitivity of these bone marrow populations between the two genotypes was less marked (not shown).

### *Rassf5*^−/−^ mice develop transplantable AML with aging

Work in our laboratory and others determined that 80% of *Irf8*^−/−^ mice develop AML (AML or BC) by ∼36 weeks of age (in a low pathogen environment; earlier in standard housing) (16, 18). If impaired Nore1 expression contributes to this, *Rassf5*^−/−^ mice might also have a tendency for leukemogenesis. To study this, we monitored aging *Rassf5*^+/−^ and *Rassf5*^−/−^ mice for circulating myeloid blasts with wildtype littermates as controls. Mice with ≥20% circulating myeloid blasts were sacrificed for further analysis. For these studies, development of AML was defined as ≥20% myeloid blasts by histology and flow cytometry in peripheral blood and bone marrow.

**Figure 5:**
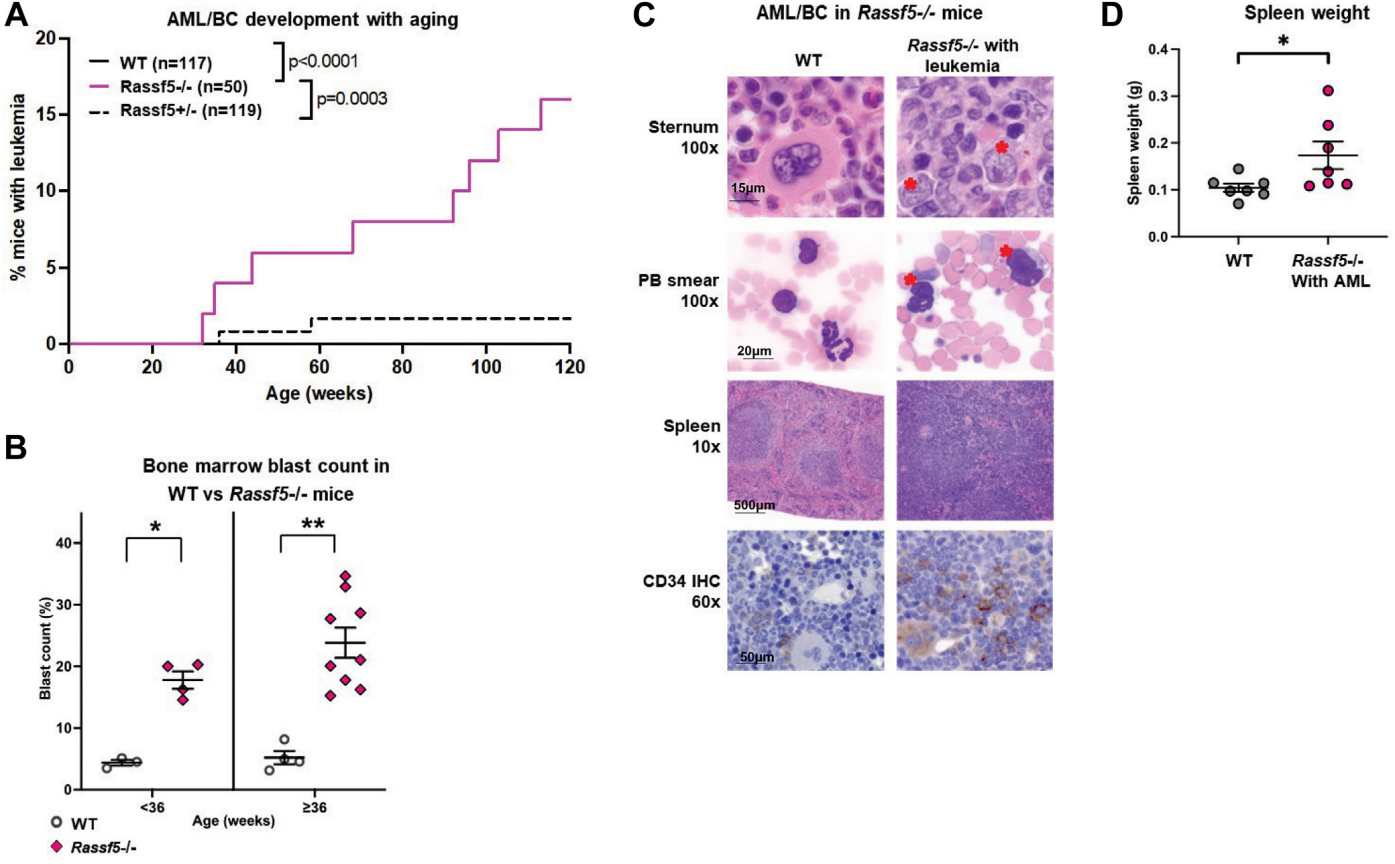
***Rassf5***^**−/−**^**mice develop acute myeloid leukemia with aging.** Aging *Rassf5*^−/−^, *Rassf5*^+/−^, and wildtype mice were compared for abundance of bone marrow myeloid blasts and spleen size. *A*, the incidence of acute myeloid leukemia (AML) is greater in *Rassf5*^−/−^ mice compared with *Rassf5*^+/−^ mice. Peripheral blood was analyzed for white blood cell counts, and mice with >20% blasts were considered to have AML. Statistical significance determined by log-rank analysis for survival curves. *B*, bone marrow blasts increase with age in *Rassf5*^−/−^ mice. Significantly more myeloid blasts, as identified by histologic examination of bone marrow biopsies, were present in young and aging *Rassf5*^−/−^ mice compared with Wt. Statistically significant differences ∗ (*p* = 0.02, n = 5) or ∗∗ (*p* = 0.001, n = 5). *C*, myeloid blasts accumulate in the peripheral blood, bone marrow, and spleen of *Rassf5*^−/−^ mice with aging. Bone marrow myeloid blasts indicated by ∗. Immunohistochemistry of sternal bone marrow reveals CD34^+^*Rassf5*^−/−^ myeloid blasts and megakaryocytes (*stained brown*). Aging wildtype mice were controls. *D*, *Rassf5*^−/−^ mice with AML develop splenomegaly. Spleen weight in *Rassf5*^−/−^ mice with AML *versus* wildtype was determined. Statistical significance indicated by ∗ (*p* = 0.01, n = 8). Error bars represent SD.

We found that 16% of *Rassf5*^−/−^ mice developed AML compared with <0.5% of *Rassf5*^+/−^ mice (*p* = 0.0007, n = 50) and none of the wildtype littermates (Fig. 5*A*). Of *Rassf5*^−/−^ mice developing AML, 75% were older than 36 weeks and 50% older than 60 weeks. Consistent with this, the mean percent of bone marrow myeloid blasts in *Rassf5*^−/−^ mice older than 36 weeks was 23.8% ± 2.4% *versus* 5.2% ± 1.1% in wildtype mice (*p* = 0.0002, n = 50) (Fig. 5*B*). In *Rassf5*^−/−^ bone marrow, cells with myeloid blast morphology were CD34^+^ by immunohistochemistry (Fig. 5*C*), in addition to being Sca1^+^ckit^+^ by flow cytometry (see later). Megakaryocytes in *Rassf5*^−/−^ bone marrow were also CD34^+^ (Fig. 5*C*). This was not observed in wildtype mice, but CD34^+^ megakaryocytes were previously described in myeloproliferative neoplasms (48). Development of AML in *Rassf5*^−/−^ mice was associated with splenic infiltration by myeloid blasts (Fig. 5*C*) and splenomegaly (*p* = 0.018, n = 7 *versus* wildtype mice) (Fig. 5*D*).

**Figure 6:**
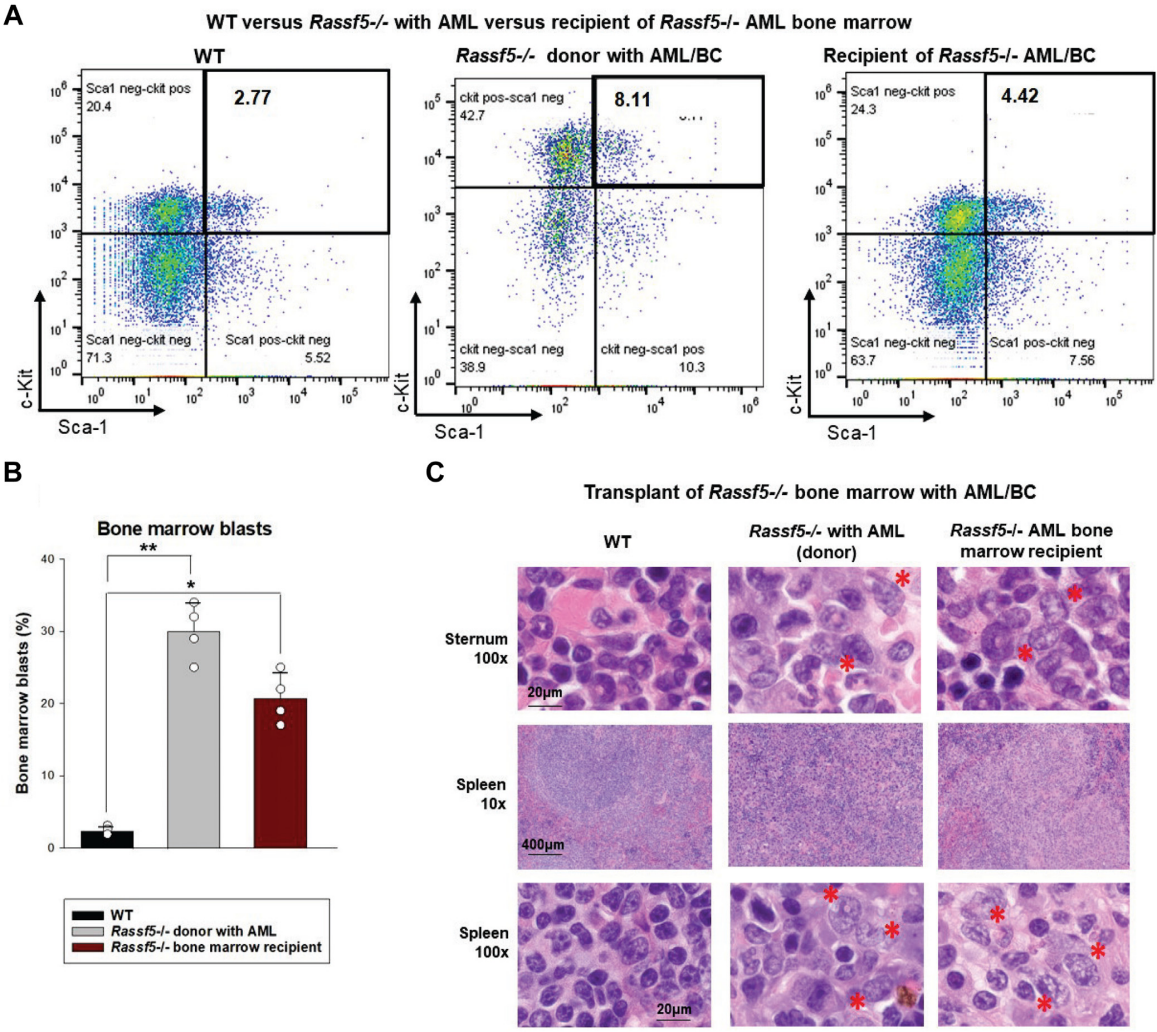
**Acute myeloid leukemia (AML) in *Rassf5***^**−/−**^**mice is transplantable.** Lin^−^ckit^+^ bone marrow cells from *Rassf5*^−/−^ mice with AML were transplanted into wildtype recipients and observed for AML (N = 2 donors). *A*, Lin^−^Sca1^+^ckit^+^ cells expand in wildtype recipients of *Rassf5*^−/−^ bone marrow as AML develops. Bone marrow Lin^−^ cells were assessed for Sca1 and ckit by flow cytometry. *B*, bone marrow myeloid blasts are identified post transplantation in wildtype recipients of *Rassf5*^−/−^ bone marrow. Comparison was made to age-matched wildtype controls. Statistical significance is indicated by ∗ (*p* = 0.03, n = 4) or ∗∗ (*p* = 0.0006, n = 4). Error bars represent SD, and *open circles* represent individual data points. *C*, myeloid blasts accumulate in the bone marrow and spleen of wildtype recipients of *Rassf5*^−/−^ AML bone marrow. Myeloid blasts are indicated by ∗.

To further characterize the disorder that developed in aging *Rassf5*^−/−^ mice, we transplanted sublethally irradiated wildtype mice with bone marrow from *Rassf5*^−/−^ mice with AML (defined as aforementioned). *Rassf5*^−/−^ donor mice were all older than 36 weeks, and these mice had expansion of the bone marrow LSK population compared with wildtype control littermates of the same age (Fig. 6*A*). Consistent with transplantable AML, myeloid blasts rapidly appeared in the circulation, bone marrow, and spleen (Fig. 6, *B* and *C*) of all recipients. LSK cells were significantly expanded in bone marrow recipients compared with wildtype mice, also consistent with this conclusion (Fig. 6*A*).

### *Rassf5*^−/−^ HSCs accumulate DNA damage with aging

**Figure 7:**
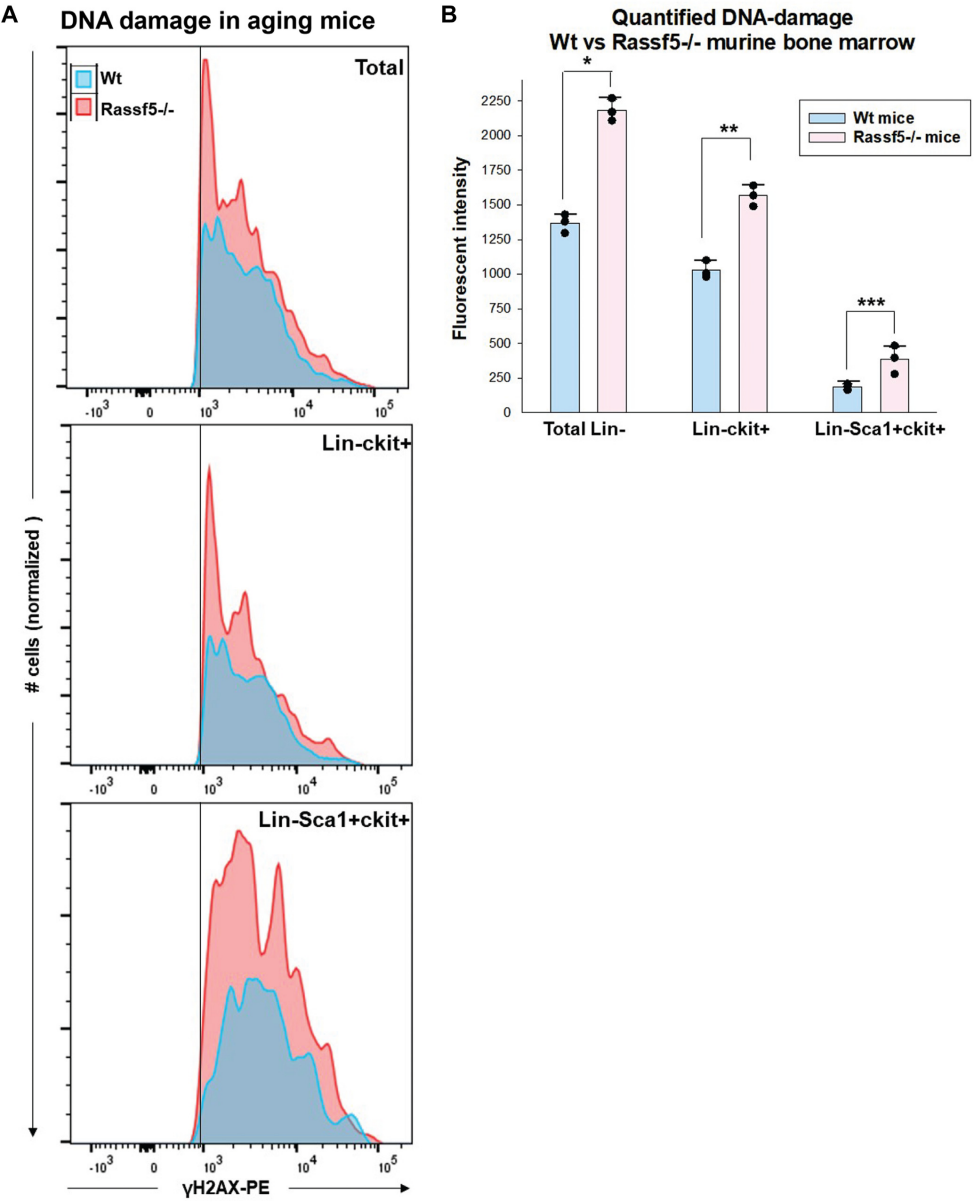
**DNA damage in aging *Rassf5***^**−/−**^**murine bone marrow stem and progenitor cells is greater than in aging wildtype mice.** Bone marrow populations from *Rassf5*^−/−^ and wildtype mice >42 weeks of age were analyzed for accumulation of DNA damage (n = 4 per group) by flow cytometry for γH2AX as a measure of double-stranded DNA damage. *A*, Rass5^−/−^ bone marrow Lin^−^, Lin^−^ckit^+^, and Lin^−^Sca1^+^ckit^+^ had more DNA damage than wildtype cells. Fluorescent intensity was plotted as a function of cell number. *B*, quantification reveals damage events are significantly more frequent in cell populations from *Rassf5*^−/−^ mice compared with wildtype. Area under the curve was calculated to compare DNA damage. Statistically significant differences are indicated by ∗ (*p* = 0.002), ∗∗ (*p* = 0.003), or ∗∗∗ (*p* = 0.001). Error bars represent SD, and *black circles* represent individual data points.

Development of AML in *Rassf5*^−/−^ mice suggests that an age-associated increase in apoptosis resistance of LSK cells permits accumulation of DNA damage, eventually leading to transformation. To investigate this, we analyzed the bone marrow of 40- to 45-week-old *Rassf5*^−/−^ and wildtype mice. Although none of the *Rassf5*^−/−^ mice in this experiment had overt AML, the percent of bone marrow myeloid blasts was significantly greater than in comparably aged wildtype mice (12.4% ± 1.5% *versus* 3.6% ± 1.0%, *p* = 0.04, n = 4). We assessed total Lin^−^ bone marrow cells, LK and LSK cells from the two genotypes by flow cytometry for γH2AX staining as a marker for double-stranded DNA damage (assessing fluorescent intensity as a function of cell number) (Fig. 7*A*). We found significantly more DNA damage in aging *Rassf5*^−/−^ mice compared with wildtype in each of these populations by this assay (expressed as area under the curve, *p* ≤ 0.003 for comparison of genotypes in the three tested populations) (Fig. 7*B*).

**Figure 8:**
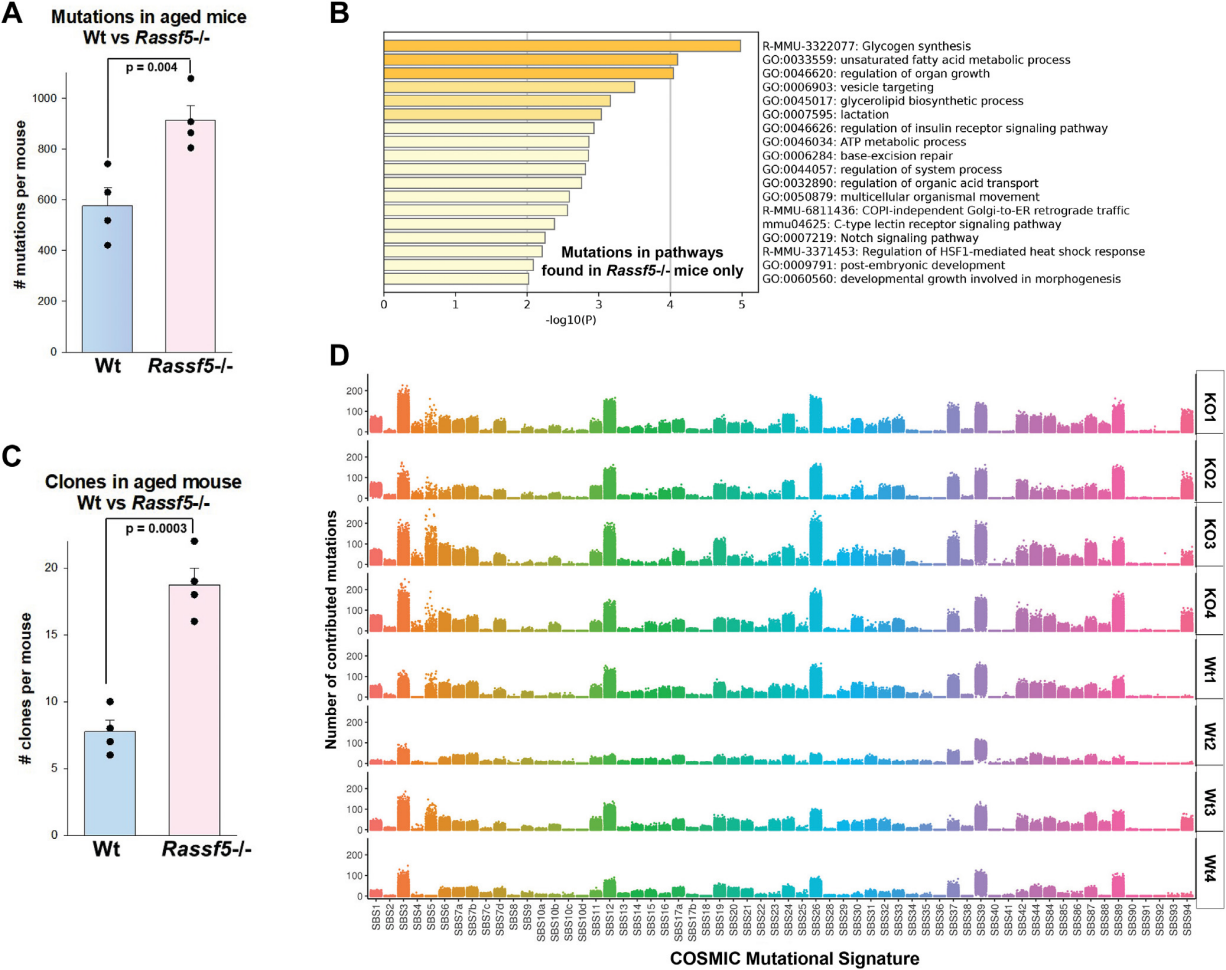
**Whole exome sequencing (WES) demonstrates a relative increase in mutations with aging in bone marrow cells from *Rassf5***^**−/−**^**mice compared with wildtype mice.** Total bone marrow mononuclear cells from *Rassf5*^−/−^ or wildtype mice were subjected to WES (n = 4 per group). *A*, the number of mutations is greater in the bone marrow of *Rassf5*^−/−^ mice compared with wildtype. Total numbers of mutations per mouse were quantified. Error bars represent SD, and *black circles* represent individual data points. *B*, Gene Ontology analysis reveals mutations in pathways involved in protein metabolism and DNA repair in *Rassf5*^−/−^ mice *versus* wildtype. Mutations identified in *Rassf5*^−/−^ and wildtype mice were compared. *C*, *Rassf5*^−/−^ mice had a higher incidence of clonal expansion compared with wildtype. Variant allele frequency (VAF) was calculated for identified mutations in both genotypes. VAF >40% was considered a clone. Error bars represent SD, and *black circles* represent individual data points. *D*, Catalogue of Somatic Mutations in Cancer (COSMIC) mutation signatures in *Rassf5*^−/−^ and wildtype mice are divergent. Mutation patterns specific to aging *Rassf5*^−/−^ mice are consistent with defective DNA repair. Individual *Rassf5*^−/−^ mice are indicated as KO1–4 and wildtype mice as Wt1–4.

As another approach to exploring mutagenesis in aging *Rassf5*^−/−^ *versus* wildtype control littermates, we analyzed genomic DNA from total bone marrow mononuclear cells by whole exome sequencing. For these studies, we quantified the average number of single base pair mutations, deletions, or insertions per mouse in aged cohorts. Consistent with flow cytometry data, we identified a significantly higher incidence of mutations in aging *Rassf5*^−/−^ mice compared with comparably aged wildtype mice (*p* = 0.004, n = 4) (Fig. 8*A*). We identified a common set of genes that were mutated in both aging *Rassf5*^−/−^ and wildtype littermate cohorts and may be characteristic of aging in this strain. However, we also identified a set of mutations unique to *Rassf5*^−/−^ mice. By Gene Ontology analysis, *Rassf5*^−/−^ specific mutations were in pathways related to nucleotide base excision repair, Notch signaling, ATP metabolism, and synthesis of glycogen and glycerolipids (Fig. 8*B*); pathways with potential to impact leukemogenesis or sustained inflammation.

In human subjects, clonal hematopoiesis of indeterminant potential is defined by a VAF of >2% for a mutation (1). Therefore, we analyzed VAF for point mutations in aging *Rassf5*^−/−^ mice or wildtype control littermates that we identified by whole exome sequencing. To ensure these mutations represented cell populations with relative expansion, we focused on clones with VAF of >20%. We identified a significant excess of mutations meeting this definition in aging *Rassf5*^−/−^ mice compared with wildtype littermate controls (*p* = 0.0003, n = 4) (Fig. 8*C*). All genes with VAF >20% in aging wildtype mice were also mutated in *Rassf5*^−/−^ mice, but there was set of genes with variant alleles more specific to aging *Rassf5*^−/−^ mice. This included *Nup98* (present in all three of four *Rassf5*^*−/−*^ mice), *Fam43a*, *Usp47*, and *Fam181a*.

We analyzed mutational signatures in aging *Rassf5*^−/−^ and wildtype littermate control mice using the Catalogue of Somatic Mutations in Cancer (COSMIC) database (Fig. 8*D*) (49). In *Rassf5*^−/−^ mice, signatures were enriched for pathways associated with defective homologous recombination repair (SBS26 and SBS44), defective DNA mismatch repair (SBS3), and an age related clock-like signature (SBS5). These patterns were consistent with pathway analysis of mutations specific to aging *Rassf5*^−/−^ murine bone marrow, aforementioned.

### Nore1 is required to terminate of emergency granulopoiesis

We previously demonstrated enhanced and sustained alum-induced emergency granulopoiesis in *Irf8*^−/−^ mice (18). Repetition every 4 weeks resulted in AML in 50% of these mice after two alum injections, and shortened survival of *Irf8*^−/−^ mice compared with steady state (18). If Nore1 contributes to termination of emergency granulopoiesis, we anticipated a similar phenotype in *Rassf5*^−/−^ mice. Therefore, we injected *Rassf5*^−/−^ every 4 weeks with alum to induce emergency granulopoiesis or saline as a steady-state control. Wildtype littermates and *Irf8*^−/−^ mice were controls in these studies. Peripheral blood counts were analyzed every 2 weeks.

**Figure 9:**
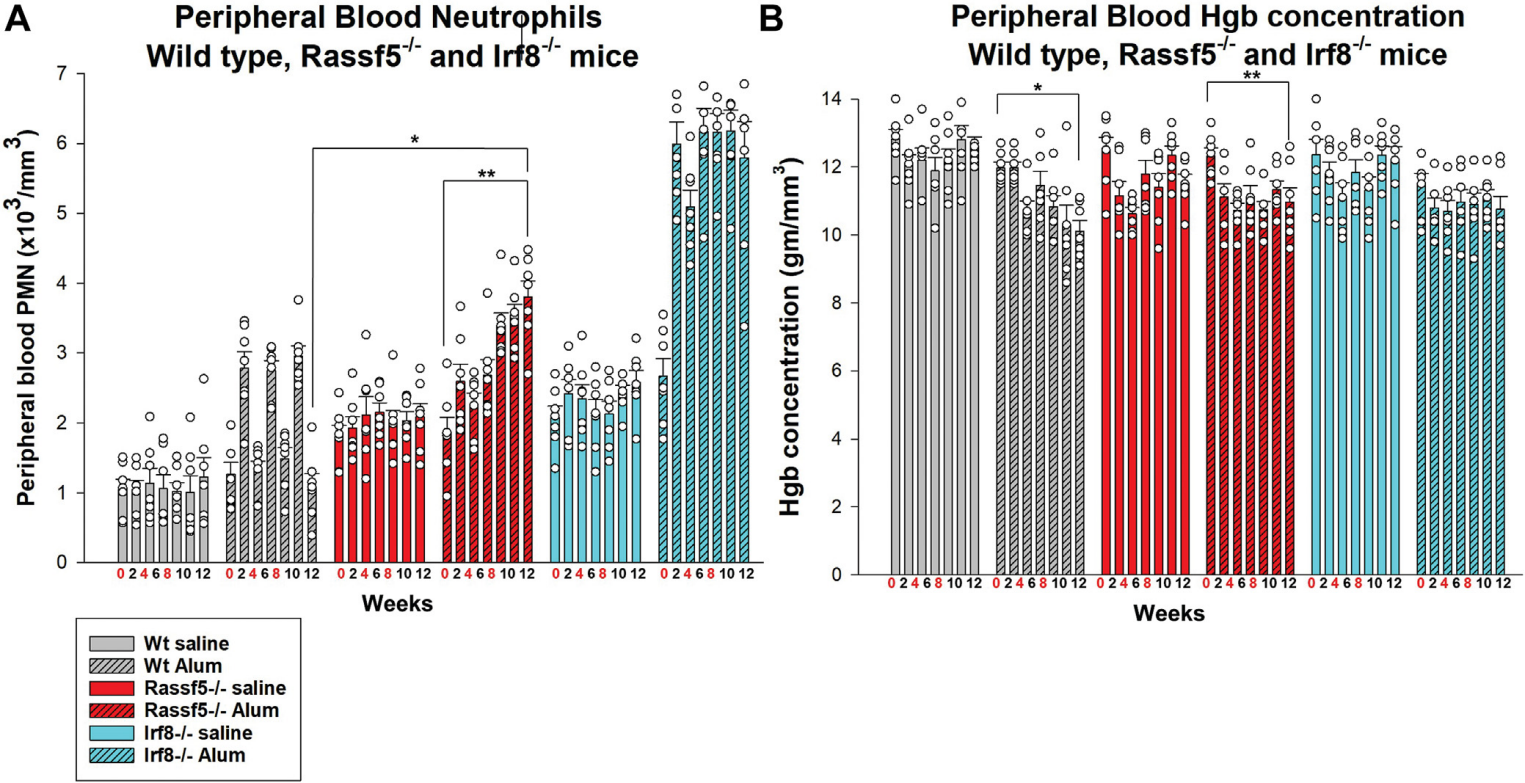
**Nore1 is required for termination of emergency granulopoiesis.***Rassf5*^−/−^, *Irf8*^*−/−*^, and wildtype mice were injected with alum to stimulate Nlrp3-induced emergency granulopoiesis or saline as a steady-state granulopoiesis control (injection weeks indicated in *red*). Peripheral blood counts were determined every 2 weeks. *A*, *Rassf5*^−/−^ mice exhibit progressive neutrophilia with repeated emergency granulopoiesis episodes. The increase in circulating neutrophils is enhanced and sustained in *Rassf5*^−/−^ and *Irf8*^*−/−*^ mice after alum injections compared with wildtype mice. The differences are more exaggerated in *Irf8*^*−/−*^ mice. Statistically significant differences indicated by ∗ (*p* = 0.01) or ∗∗ (*p* = 0.0004). *B*, mild anemia develops in the three genotypes during repeated alum-induced emergency granulopoiesis episodes. Statistically significant differences indicated by ∗ (*p* = 0.0009) or ∗∗ (*p* = 0.02). At least six mice per group were analyzed for the panels in this figure, and error bars represent SD and *open circles* represent individual data points. Irf8, interferon regulatory factor 8.

In alum-injected wildtype mice, peripheral blood neutrophilia was maximal at 2 weeks, and steady state resumed by 4 weeks, as in our prior studies (Fig. 9*A*) (18, 19). In contrast, neutrophils did not return to baseline abundance in *Rassf5*^−/−^ mice by 4 weeks post alum injection (*p* = 0.0007, n = 7 *versus* steady state) (Fig. 9*A*). Peak neutrophilia increased with each alum injection in *Rassf5*^−/−^ mice and was significantly greater than wildtype mice by the third injection (*p* = 0.001, n = 7). However, starting with the first alum injection, aberrant neutrophilia was less profound in *Rassf5*^−/−^ mice compared with *Irf8*^−/−^ mice (*p* = 0.0004, n = 7 for the last injection). Repeated alum injection induced comparable and mild anemia in all three genotypes (*p* ≤ 0.02, n = 7) (Fig. 9*B*). Circulating monocytes were not significantly different in *Rassf5*^−/−^ mice compared with wildtype during emergency granulopoiesis, and platelet counts did not vary significantly during this experiment of any of the three genotypes (not shown).

**Figure 10:**
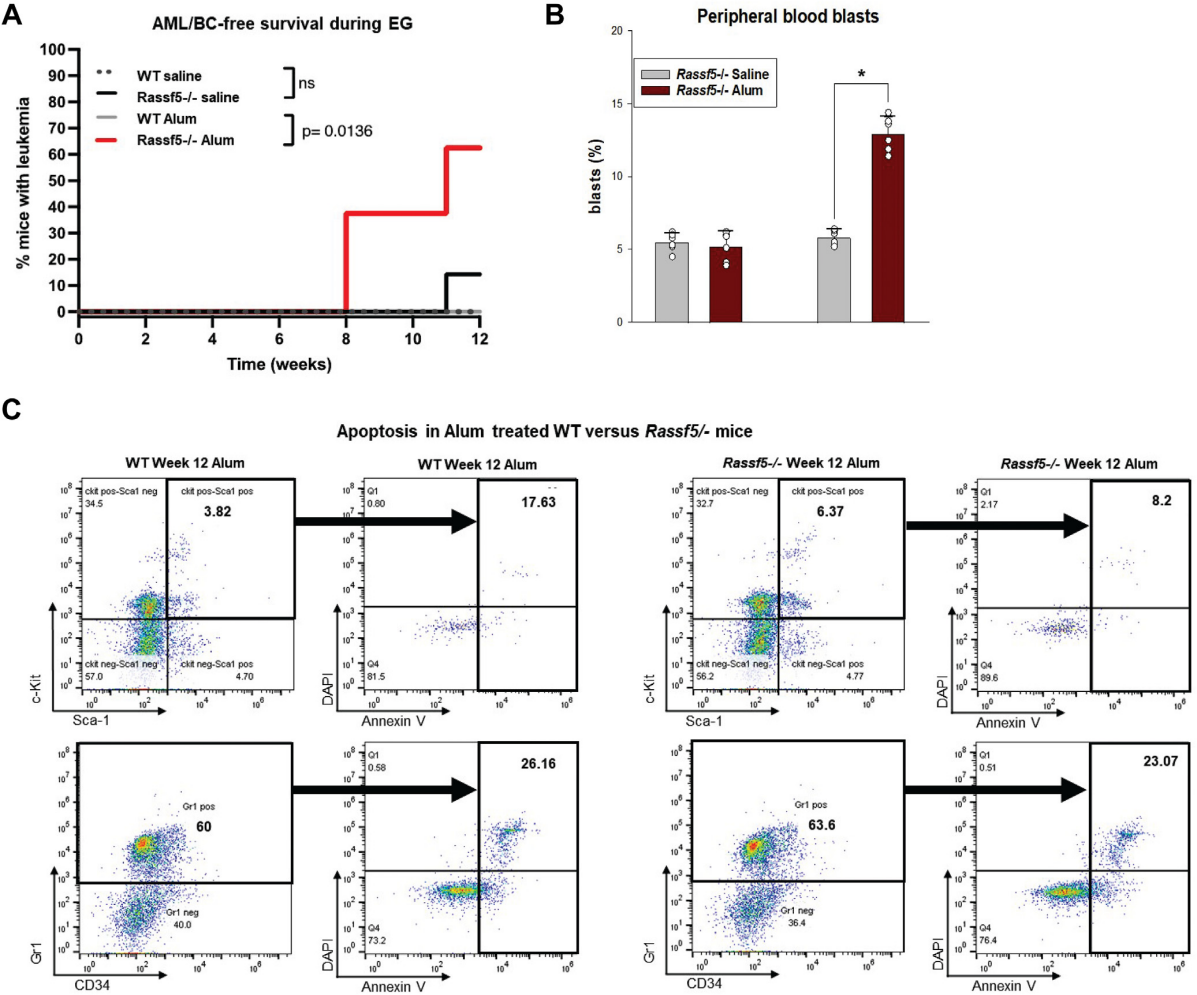
**Episodes of emergency granulopoiesis accelerate development of acute myeloid leukemia (AML) in *Rassf5***^**−/−**^**mice.***Rassf5*^−/−^ and wildtype mice were injected every 4 weeks with alum to stimulate emergency granulopoiesis or saline as a steady-state control. Peripheral blood and bone marrow were analyzed for AML/BC (myeloid blasts >20%). At least six mice per group were analyzed. *A*, repeated episodes of emergency granulopoiesis significantly increase the number of *Rassf5*^−/−^ mice developing AML compared with steady state. Statistical significance was determined by log-rank analysis. Nonsignificant values indicated by “ns.” *B*, circulating myeloid blasts increase at earlier time points in alum-injected *Rassf5*^−/−^ mice compared with *Rassf5*^−/−^ mice at steady state. Mice were studied at weeks 8 and 12. Statistical significance indicated by ∗ (*p* = 0.03). Error bars represent SD, and *open circles* represent individual data points. *C*, Lin^−^Sca1^+^ckit^+^ cells in the bone marrow of alum-injected *Rassf5*^−/−^ mice were relatively expanded (*p* = 0.02) and apoptosis resistant (*p* = 0.03) compared with wildtype cells. Gr1^+^ bone marrow cells are not significantly different between the two genotypes (*p* = 0.45). Mice were analyzed at 8 weeks. BC, blast crisis.

In *Rassf5*^−/−^ mice, we found that repeated episodes of emergency granulopoiesis enhanced development of AML (*i.e.*, BC) compared with the same duration at steady state (Fig. 10*A*). Specifically, 62.5% of *Rassf5*^−/−^ mice had AML after the third alum injection compared with 14.3% of saline-injected *Rassf5*^−/−^ mice at this time point. Consistent with this, the mean number of circulating myeloid blasts in *Rassf5*^*−/−*^ mice after three alum injections was 12.7% ± 2.19% compared with 6.68% ± 1.28% in saline-injected controls (*p* = 0.03, n = 8) (Fig. 10*B*).

Eight weeks after the second alum injection, the total number of bone marrow mononuclear cells was greater in *Rassf5*^−/−^ mice relative to alum-injected wildtype mice (6.69 × 10^6^ ± 3.58 × 10^6^ cells *versus* 3.75 × 10^6^ ± 5.41 × 10^6^ cells, *p* = 0.005, n = 3). LSK bone marrow cells were expanded at this time point in alum-injected *Rassf5*^−/−^ mice but not in wildtype (*p* = 0.04, n = 3) (Fig. 10*C*). However, the Gr1^+^ population was not expanded in alum-injected *Rassf5*^−/−^ mice *versus* wildtype, consistent with differentiation block and evolving AML in the former. Expansion of LSK cell in *Rassf5*^−/−^ murine bone marrow during multiple emergency granulopoiesis episodes was associated with decreased apoptosis compared with cells from wildtype littermate control mice under the same conditions (*p* = 0.03, n = 3) (Fig. 10*C*). Apoptosis was not significantly different in Lin^+^Gr1^+^ cells from alum-injected *Rassf5*^−/−^ *versus* wildtype mice (*p* = 0.45, n = 3) (Fig. 10*C*).

### Nore1a contributes to apoptosis resistance of *Irf8*^−/−^ bone marrow cells

**Figure 11:**
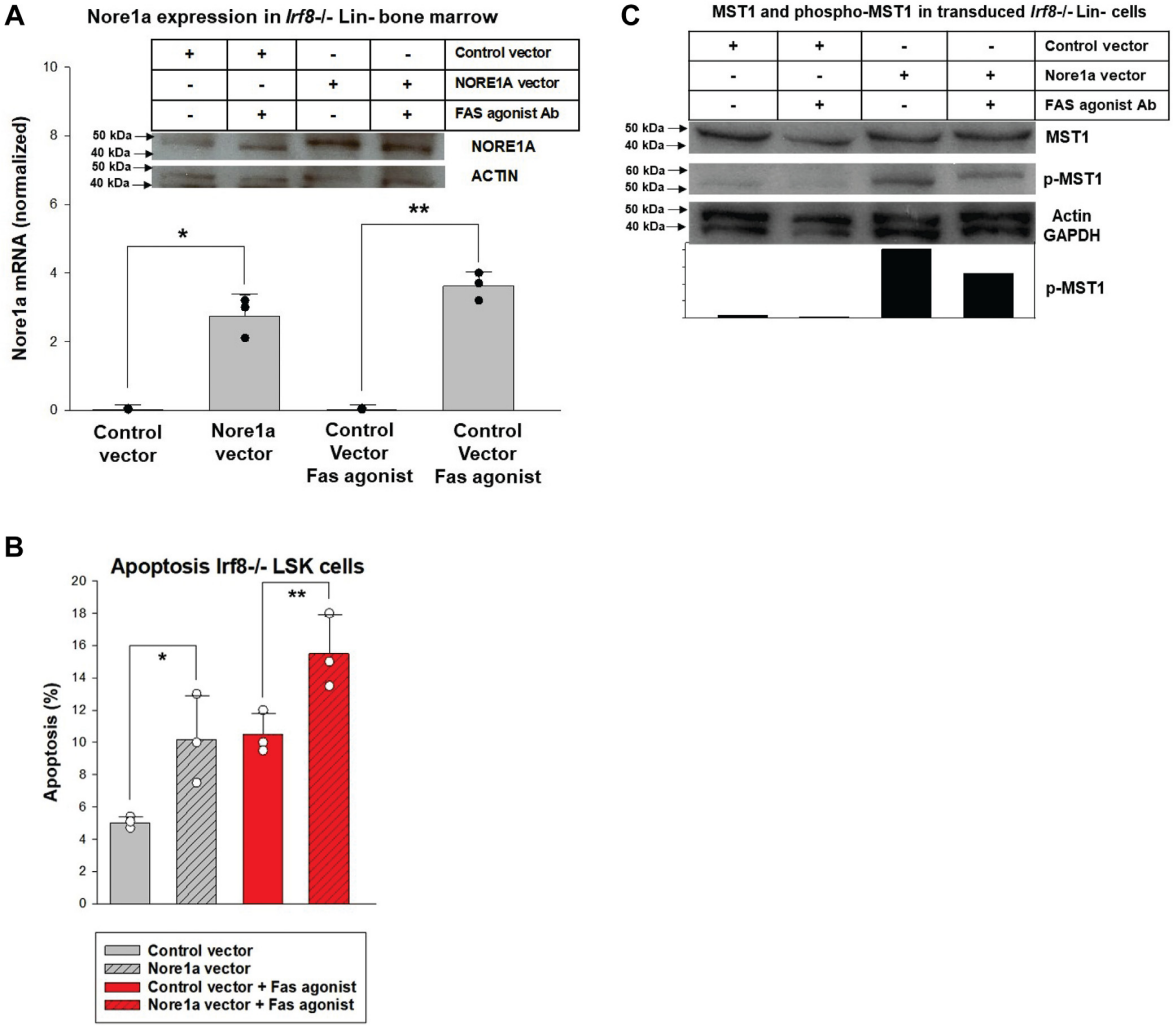
**Nore1a re-expression enhances apoptosis of *Irf8***^***−/−***^**Lin**^**−**^**Sca1**^**+**^**ckit**^**+**^**(LSK) cells.** Bone marrow from *Irf8*^*−/−*^ mice was transduced with a retroviral vector to express Nore1a or empty control vector. *A*, Nore1a expression is greater in transduced *Irf8*^*−/−*^ cells. Expression of Nore1 was confirmed by Western blot and quantitative real-time PCR. Significant differences indicated by ∗ (*p* = 0.002, n = 3) or ∗∗ (*p* = 0.0004, n = 3). Error bars represent SD, and *black circles* represent individual data points. *B*, Nore1a re-expression increases Fas-induced apoptosis in *Irf8*^*−/−*^ LSK cells. Nore1a transduction also enhanced intrinsic apoptosis in *Irf8*^*−/−*^ LSK cells. Statistical significance indicated by ∗ (*p* = 0.03, n = 3) or ∗∗ (*p* = 0.007, n = 3). Error bars represent SD, and *open circles* represent individual data points. *C*, Nore1a re-expression in *Irf8*^*−/−*^ cells increases phospho-Mst1 but not total Mst1 protein. Western blots were probed for actin and GAPDH as loading controls. Irf8, interferon regulatory factor 8.

In prior studies, we determined that Irf8 enhances Fas-dependent and -independent apoptosis (6, 9, 10). To investigate the contribution of Nore1a, we transduced *Irf8*^−/−^ murine bone marrow cells with a vector to express Nore1a or empty control vector (Fig. 11*A*). Since *Rassf5*^−/−^LSK cells were relatively apoptosis resistant, we quantified apoptosis in transduced Lin^−^ cells. We found Nore1a re-expression enhanced Fas-induced apoptosis in *Irf8*^−/−^LSK cells (15.65% ± 1.95% *versus* 9.94% ± 0.41%, *p* = 0.007, n = 3) (Fig. 11*B*). Intrinsic apoptosis was also increased by Nore1a expression in *Irf8*^−/−^LSK cells (10.40% ± 2.4% *versus* 5.71% ± 0.63%, *p* = 0.03, n = 3) (Fig. 11*B*).

Other investigators found that Mst1 activation by Nore1 facilitated Fas-induced apoptosis in hepatocytes (29, 30). Consistent with this, we found transduction of *Irf8*^−/−^LSK cells with a Nore1a expression vector increased phospho (activated)-Mst1 but not total Mst1 (Fig. 11*C*). Treatment with Fas agonist slightly decreased phospho-Mst1 in Nore1a-transduced *Irf8*^−/−^ cells; however, these cells were undergoing apoptosis at a higher rate than untreated cells.

## Discussion

Although Nore1a was not previously known to play a role in hematopoiesis, our current study implicated it in leukemia suppression, termination of emergency granulopoiesis, and aspects of bone marrow aging such as myeloid skewing and clonal hematopoiesis. In this work, we found that Irf8 interacted with, and activated, an Ets/Irf-binding consensus sequence in the proximal *RASSF5A* promoter. We also found expansion, apoptosis resistance, and mutagenesis in LSK cells from aging *Rassf5*^−/−^ murine bone marrow compared with wildtype controls. This was associated with myeloid skewing, clonal hematopoiesis, and predisposition to AML. During episodes of Nlrp3 inflammasome–induced emergency granulopoiesis, we found that Nore1a increased in an Irf8-dependent manner in hematopoietic stem and progenitor cells. In contrast, the *RASSF5B* promoter did not bind Irf8, and Nore1b did not exhibit Irf8-regulated expression in myeloid cells; consistent with the previously described lymphoid restriction of this isoform. Both Nore1a and b isoforms are disrupted in *Rassf5*^−/−^ mice, and these mice did not exhibit the age-associated decrease in circulating lymphocytes observed in wildtype mice. The latter is a topic of interest for future studies.

We found that loss of either Irf8 or Nore1 enhanced neutrophil production and predisposed to accumulation of mutations with aging that favor differentiation block and AML (16). This has implications for understanding mechanisms involved in age-related myeloid skewing and CHIP in human subjects (1). It is not clear how some commonly identified CHIP-associated mutations facilitate leukemogenesis, and mechanisms for variable development of AML in subjects with the same mutation are also undefined. We hypothesize that cooperation of such leukemia-associated mutations with an age-associated decrease in Irf8, and thereby Nore1a, may define such mechanisms. For example, we found a higher incidence of *Nup98* point mutations in aging *Rassf5*^−/−^ mice compared with wildtype littermate controls. These mutations were present at a VAF that suggested clonal expansion of mutant cells. Nup98 is involved in gene transcription and plays a key role in mRNA transport from the nucleus (50). Chromosomal translocations involving *NUP98* port

Compared with *Irf8*^−/−^ mice, AML occurred in *Rassf5*^−/−^ mice after a longer lag time and with a lower incidence, even during episodes of emergency granulopoiesis. This is not surprising, since Irf8 also influences target genes involved in cytokine-induced proliferation, DNA repair, and proapoptotic mechanisms that are not attributable to Nore1. However, consistent with a contribution of decreased Nore1a to apoptosis resistance of *Irf8*^−/−^ cells, Nore1a re-expression facilitated Fas-induced and intrinsic apoptosis in HSC and myeloid progenitor cells. Apoptosis was most rapid in *Irf8*^−/−^ cells with the highest Nore1a expression level, leading to an underestimation of the effect. However, transducing *Irf8*^−/−^ cells with a Nore1a expression vector activated Mst1, a known downstream target.

We found that relative apoptosis resistance of LSK cells from aging *Rassf5*^−/−^ mice was associated with enhanced accumulation of DNA damage compared with aging wildtype mice. By whole exome sequencing, we defined a mutation profile unique to aging *Rassf5*^−/−^ bone marrow *versus* aging wildtype littermate controls. This included impaired base excision repair and DNA mismatch repair; deficiencies anticipated to permit accumulation of DNA damage in apoptosis-resistant *Rassf5*^−/−^ cells. An increase in bone marrow neutrophils in aging *Rassf5*^−/−^ mice may contribute excess reactive oxygen species to the microenvironment *via* activation of the phagocyte NADPH oxidase. The combination of increased reactive oxygen species, decreased apoptosis, and impaired DNA repair would favor mutagenesis leading to clonal hematopoiesis and/or AML in aging *Rassf5*^−/−^ mice. Nore1a may also contribute to cell cycle arrest and regulate Mdm2 (32, 51, 52, 53, 54). The contribution of Nore1 to Mdm2/Tp53-induced cell cycle pause and/or apoptosis during the DNA-damage response is of interest for future study.

We found that *Rassf5*^−/−^ mice were unable to efficiently terminate an emergency granulopoiesis response, similar to *Irf8*^−/−^ mice and a murine model of CML (18, 19). This suggests that Nore1a enhances apoptosis in LSK cells to contribute to emergency granulopoiesis termination. Apoptosis of *Rassf5*^−/−^Lin^+^Gr1^+^ cells was not impaired, suggesting differentiation stage specificity to regulation of the innate immune response by Nore1a. We hypothesize enhanced genotoxic stress during emergency granulopoiesis, coupled with apoptosis resistance of LSK cells, accelerated accumulation of mutations in *Rassf5*^*−/−*^ mice. This has implications for understanding the contribution of infectious challenges to clonal hematopoiesis in aging human bone marrow with decreased Irf8 expression.

**Figure 12:**
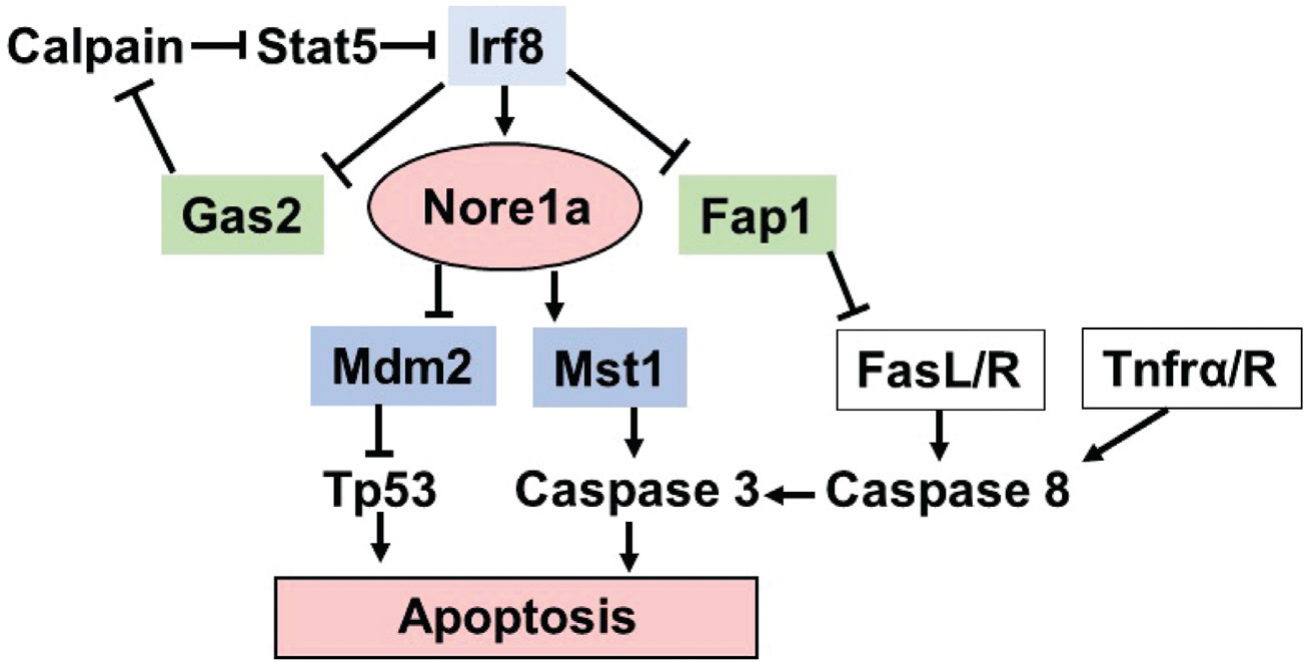
**Schematic representation of interactions between Nore1a, upstream regulators, and downstream effectors of apoptosis.** Irf8 modulates apoptosis by enhancing Nore1a expression but repressing expression of Fap1 (a Fas antagonist) and Gas2 (a calpain inhibitor). In the context of apoptotic signals such as Fas and TNF-α, Nore1a interacts with downstream targets to mediate apoptosis. Irf8, interferon regulatory factor 8; TNFα, tumor necrosis factor alpha.

In some solid tumors, *RASSF5* is inactivated by promoter hypermethylation or chromosomal translocation (25, 32, 33, 34, 35, 36, 37, 38, 39, 40, 41, 42, 43, 44, 45). Here, we define another mechanism for decreased *RASSF5* transcription; decreased Irf8 expression as found in myeloid leukemias or with aging. Mst1 is a mediator of Fas or TNFα-induced apoptosis, but Nore1a may also impair cell survival *via* other interactions (Fig. 12) (29, 31, 51, 52). Further studies to identify effectors downstream from Nore1a are the topic of ongoing work.

## Experimental procedures

### Plasmid vectors and myeloid cell line transfections

Irf8 cDNA (also referred to as Icsbp) was obtained from Dr Ben Zion-Levi (Technion) and subcloned into the mammalian expression vector pcDNAamp (Stratagene). Irf8-specific shRNA and scrambled control sequences were designed using the Promega website (Promega). Prior published work documented Irf8 overexpression or knockdown in U937 cells by these vectors (6, 9, 10). The Nore1a cDNA was subcloned into a pMSCVneo retroviral vector (Stratagene). Sequences from the *RASSF5A* or *B* promoters were generated by PCR from the U937 myeloid cell line. PCR products were sequenced and compared with genomic databases. *RASSF5* promoter *A*/reporter constructs were generated by subcloning 2 kb, 250 bp, 180 bp, and 110 bp of 5′ flank into the pGL3-enhancer reporter vector (Promega). *RASSF5B* constructs with 2 kb and 580 bp of 5′ flank were also generated. A minimal promoter–reporter construct was generated by subcloning a double-stranded oligonucleotide with three copies of −180 bp to −157 of *Rassf5* promoter A into pGL3-promoter reporter vector (Promega) (TTCTCTTGGGTCGT CCTTCCGCC, Ets/Irf consensus underlined). The oligonucleotide was custom synthesized by Integrated DNA Technologies.

U937 myeloid leukemia cells (obtained from Dr Andrew Kraft, University of Arizona) were maintained in Dulbecco's modified Eagle's medium, 10% fetal bovine serum (FBS), and 1% penicillin–streptomycin. Cells (30 × 10^6^/ml) were transfected by electroporation with vectors to overexpress or knockdown Irf8 (or relevant control vectors), *RASSF5* promoter reporter constructs (or empty reporter vector control), and a CMV/renilla-luciferase reporter (to control for transfection efficiency). Dual luciferase assays were performed per manufacturer’s instructions (Promega). Reporter activity for the empty control vectors was subtracted from the activity from vectors with *RASSF5* sequences as background.

### Chromatin immunoprecipitation

U937 cells were incubated briefly in formaldehyde-supplemented media followed by sonication to generate chromatin fragments of ∼0.5 kb. Lysates were immunoprecipitated with rabbit anti-Irf8 serum or preimmune serum (Covance, Inc), and immunoprecipitated chromatin was hybridized to a CpG island microarray, as described (6). Significant CpG island precipitation was determined by nearest neighbor normalization, as described, and was at least threefold (6). Experiments were performed in triplicate, and only genes identified by in all three independent experiments were further investigated.

Chromatin immunoprecipitation was also performed with murine bone marrow cells and antibodies to Irf8, Tri-Methyl K4 Histone H3 (Cell Signaling Technology, Inc), or irrelevant antibody (immunoglobulin G isotype control). Specific precipitation of the *Rassf5* promoter *A* sequence was determined by quantitative PCR. Primers designed to flank an Ets/Irf-binding consensus sequence in the promoter were *A* forward (5′-ACTCCTAAATCCACGCGGC-3′) and *A* reverse (5′-ATCTAGGAGCAGCGGGTAGG-3′). Primers were also designed to flank a sequence from the first exon of *Rassf5* and the CpG island in *RASSF5* promoter B as negative controls. The experiment was performed in triplicate in three independent precipitations.

### Quantitative real-time PCR and Western blotting

RNA was isolated using the TRIzol reagent (Invitrogen) and tested for integrity by denaturing gel electrophoresis. Primers were designed with Applied Biosystems software, and PCR was performed using SYBR Green according to the “standard curve” method. Results were normalized to actin and GAPDH to control for mRNA abundance.

For Western blots, cells were lysed in SDS sample buffer, separated by SDS-PAGE, transferred to nitrocellulose, and serially probed with antibodies to Nore1 (Thermo Fisher Scientific), total Mst1 (Cell Signaling Technology), phospho-Mst (Cell Signaling Technology), or GAPDH (loading control). Each experiment was repeated with at least three different lysates, and a representative blot is shown.

### Mice, murine emergency granulopoiesis studies, and bone marrow transplant

*Rassf5*^−/−^ C57BL/6 mice (a gift from Dr Shairaz Baksh, University of Alberta) were bred with Wt C57BL/6 mice to generate *Rassf5*^+/−^ mice. *Rassf5*^+/−^ mice were used to generate *Rassf5*^−/−^ mice, *Rassf5*^+/−^ mice, and Wt litter mate control mice. Breeding pairs of *Rassf5*^+/−^ mice were crossed for >10 generations. Genotyping was performed as described (31). A similar strategy was followed to establish breeding colonies of *Irf8*^+/−^ C57BL/6 mice; initially obtained from Dr Keiko Ozato (National Institutes of Health) (16). Mice were housed in a low pathogen “barrier” environment as described (18).

To induce activation of the Nlrp3 inflammasome and induce emergency granulopoiesis, mice were injected intraperitoneally with ovalbumin/aluminum hydroxide–magnesium hydroxide (*i.e.*, alum) or saline (as a steady-state control) every 4 weeks for 12 weeks, as described (18, 22). Multiple alum injections were performed to mimic repeated infectious challenges (equivalent to several episodes of infection per year in humans). Peripheral blood counts were determined every 2 weeks using tail vein phlebotomy. Mice were randomly assigned to cohorts (seven per group) by coin flip. Average blood counts for cohorts in each genotype were not significantly different at the start of the experiment. All mice in each cohort were analyzed.

In other studies, *Rassf5*^−/−^ mice with AML (bone marrow myeloid blasts ≥20%) were sacrificed, and bone marrow was harvested for transplantation studies. Sublethally irradiated wildtype C57BL/6 mice were transplanted with 0.5 × 10^5^ Lin^−^ cells. Peripheral blood counts were determined every 2 weeks.

### Analysis of murine peripheral blood and tissues

Peripheral blood was obtained by tail vein phlebotomy for complete blood counts by automated cell counter. Circulating myeloid blasts were identified by examination of May-Grünewald–Giemsa-stained peripheral blood smears by light microscopy (100× magnification; Zeiss Axioskop microscope, Zeiss Group). Slides from decalcified sternal bone marrow and paraffin-embedded spleen tissue were stained with hematoxylin and eosin by the Pathology Core Facility of the Lurie Cancer Center. Light microscopy was performed, and digital images were captured (100× magnification). Sternal myeloid blast counts were verified by hand counting at least 200 cells/high-power field × 2 separate high-power fields.

### Murine bone marrow separation and retroviral transduction

Bone marrow mononuclear cells were obtained from the femurs of *Irf8*^−/−^, *Rassf5*^−/−^, or wildtype mice for use in these studies (18, 19). Lin^−^ cells were isolated from Lin^+^ cells by antibody-based magnetic bead affinity chromatography using a lineage depletion cocktail that includes antibodies to CD5 (T cell), B220 (B cell), CD11b (monocytes and neutrophils), Gr-1 (neutrophils and some monocytes), 7-4 (neutrophils), and Ter-119 (erythroid progenitors) (Miltenyi Biotec). In some experiments, Lin^−^ cells were further selected by affinity chromatography with antibodies to Sca1 and ckit. For other experiments, Lin^+^ cells were recovered and selected for the Gr1^+^ subpopulation.

For retroviral transduction experiments, Lin^−^ cells were separated as aforementioned and cultured (2 × 10^5^ cells/ml) in Dulbecco’s modified Eagle’s medium, 10% FBS, 1% penicillin–streptomycin with 10 ng/ml granulocyte–macrophage colony-stimulating factor, 10 ng/ml IL-3, and 100 ng/ml stem cell factor (R&D Systems). Cells were transduced by incubation with retroviral supernatant (∼10^7^ plaque-forming unit/ml), supplemented with polybrene (6 μg/ml), and selected for 48 h in gentamicin. Transgene expression was confirmed by PCR.

For retroviral production, 293T cells were transfected by electroporation with Nore1a/MSCVneo plasmid or control MSCVneo, supernatants were collected 48 h post transfection, and virus was titrated in NIH3T3 cells, as described (10). Cell lines were obtained from American Type Culture Collection, and all lines were validated annually by genomic fingerprinting (ATCC Whatman FTA Human STR Kit).

### Flow cytometry, apoptosis, and DNA-damage assays

Cells were washed in phosphate-buffered saline with 1% FBS, counted, and labeled with antimouse FITC-conjugated antibodies to Sca1 (Ly-6A/E) or allophycocyanin-conjugated antibodies to c-kit or Gr1 (Ly-6G/C) (Invitrogen). For some studies, apoptosis was induced by treatment with 5 μg/ml Fas-agonist antibody (mouse anti-CD95; BD Biosciences) for 16 h. Apoptosis was assessed using the Annexin V-PE apoptosis detection kit I (BD Biosciences) with 4′,6-diamidino-2-phenylindole counterstaining. Experiments were performed in triplicate.

### Whole exome sequencing and data analysis

Total bone marrow mononuclear cells were isolated from the femurs of *Rassf5*^−/−^ mice or *Rassf5*^+/+^ littermates, and DNA libraries were prepared with the Illumina TruSeq Exome Library Prep Kit. After validation with Qubit and Agilent Bioanalyzer, DNA libraries with unique barcoding indexes were pooled and hybridized to exome oligo probes to capture the exonic regions of the genome. Libraries were validated with Qubit quantification and Bioanalyzer quality check using a high-sensitivity DNA chip. Library sequencing was conducted on an Illumina NovaSeq NGS System. Paired end 150 bp reads were generated.

Read quality, in FASTQ format, was evaluated using FastQC (Illumina, version 0.11.7). Trimmed reads were aligned to the mouse genome (mm10) using the Burrows Wheeler Aligner (version 0.7.12) (54). Resulting alignment files were cleaned, sorted, and marked for duplicates using the Picard Tools (version 1.85) CleanSam, SortSam, and Mark Duplicates, respectively. Files were further filtered using the TruSeq_Exome_TargetedRegions_v1.2.bed file. Variants were detected from the processed files using bcftools mpileup with minimum base quality and maximum depth parameters set to 20 and 1000, respectively. Files were filtered for quality and depth of 20 and 10, respectively (55, 56). The resulting variant files in Variant Call Format were annotated using SnpEff (an open source tool), version 4.3. VAF was calculated as the ratio of allele depth (variant)/median depth (minimum read depth of 30). The mutational patterns analysis was done using MutationalPatterns package on R (49).

### Statistics

Statistical significance was determined by Student's *t* test (for comparison of two conditions) or ANOVA (for comparison of more than two conditions) using GraphPad Prism software (GraphPad Software, Inc) or SigmaPlot software (Systat Software Inc). Results are reported as the mean ± SD with *p* < 0.05 considered significant. Survival curves were compared by the log-rank test.

### Study approval

Animal studies were performed according to protocols approved by the Animal Care and Use Committees of Northwestern University and Jesse Brown VA Medical Center.

## Data availability

Data are available from E. Eklund (e-eklund@northwestern.edu).

## Conflict of interest

The authors declare that they have no conflicts of interest with the contents of this article.
